# Free fatty acids, glicentin and glucose-dependent insulinotropic polypeptide as potential major determinants of fasting substrate oxidation

**DOI:** 10.1038/s41598-021-95750-9

**Published:** 2021-08-17

**Authors:** Julia Hummel, Louise Fritsche, Andreas Vosseler, Corinna Dannecker, Miriam Hoene, Konstantinos Kantartzis, Hans-Ulrich Häring, Norbert Stefan, Jürgen Machann, Andreas L. Birkenfeld, Cora Weigert, Robert Wagner, Andreas Peter, Andreas Fritsche, Martin Heni

**Affiliations:** 1 Institute for Diabetes Research and Metabolic Diseases of the Helmholtz Center Munich at the University of Tübingen, Otfried-Müller-Str. 10, 72076 Tübingen, Germany; 2 German Center for Diabetes Research (DZD), Ingolstädter Landstraße 1, 85764 Neuherberg, Germany; 3 Department of Internal Medicine, Division of Diabetology, Endocrinology and Nephrology, Eberhard Karls University Tübingen, Otfried-Müller-Str. 10, 72076 Tübingen, Germany; 4 Institute for Clinical Chemistry and Pathobiochemistry, Department for Diagnostic Laboratory Medicine, Eberhard Karls University Tübingen, Hoppe-Seyler-Str. 3, 72076 Tübingen, Germany; 5 Department of Radiology, Section on Experimental Radiology, Eberhard Karls University Tübingen, Hoppe-Seyler-Str. 3, 72076 Tübingen, Germany

**Keywords:** Endocrinology, Medical research

## Abstract

The selection of carbohydrates or fat to generate intracellular energy is thought to be crucial for long-term metabolic health. While most studies assess fuel selection after a metabolic challenge, the determinants of substrate oxidation in the fasted state remain largely unexplored. We therefore assessed the respiratory quotient by indirect calorimetry as a read-out for substrate oxidation following an overnight fast. This cross-sectional analysis consisted of 192 (92 women, 100 men) either lean or obese participants. Following an overnight fast, the respiratory quotient (RQ) was assessed, after which a 5-point 75-g oral glucose tolerance test was performed. Unlike glucose and insulin, fasting free fatty acids (FFA) correlated negatively with fasting RQ (*p* < 0.0001). Participants with high levels of the ketone body β-hydroxybutyric acid had significantly lower RQ values. Fasting levels of glucose-dependent insulinotropic polypeptide (GIP) and glicentin were positively associated with fasting RQ (all *p* ≤ 0.03), whereas GLP-1 showed no significant association. Neither BMI, nor total body fat, nor body fat distribution correlated with fasting RQ. No relationship between the RQ and diabetes or the metabolic syndrome could be observed. In the fasting state, FFA concentrations were strongly linked to the preferentially oxidized substrate. Our data did not indicate any relationship between fasting substrate oxidation and metabolic diseases, including obesity, diabetes, and the metabolic syndrome. Since glicentin and GIP are linked to fuel selection in the fasting state, novel therapeutic approaches that target these hormones may have the potential to modulate substrate oxidation.

## Introduction

The selection of the predominant fuel source for intracellular energy generation is crucial for long-term health. Alterations are linked to a number of diseases, including obesity, diabetes and cardiovascular diseases^1–3^. The two major cellular energy substrates glucose and free fatty acids (FFA) are metabolized via glycolysis and beta oxidation, respectively, to generate acetyl-CoA. Acetyl-CoA is further metabolized and biochemical energy is generated in the mitochondria by a process known as oxidative phosphorylation^4^.

The relative amount of substrate that is oxidized over a certain period of time can be assessed non-invasively by indirect calorimetry. The respiratory quotient (RQ)—as a ratio of exhaled carbon dioxide to consumed oxygen—indicates the preferentially oxidized substrate^5^.

Several studies report that both fasting and 24-h-RQ are predictors for long-term weight gain. High fasting as well as average 24-h-RQ, which are indicative of predominant glucose oxidation relative to fatty acids, is linked to subsequent body fat accumulation^6–9^. The importance of altered substrate oxidation is further emphasized by the association shown between higher RQ and insulin resistance^10^, hepatic steatosis^11^, hypertension^12^ and subclinical carotid atherosclerosis^13^ in individuals with overweight/obesity.

Metabolic flexibility describes the ability to adapt substrate oxidation to substrate availability^14^. This was initially reported for the transition from the fasting to the fed state. Only later was the crucial switch of fuel oxidation in response to overnight fasting brought into focus^15^. To date, several clinical conditions with impaired metabolic flexibility have been identified. In patients with insulin-resistance, obesity and diabetes, diminished fat oxidation during nocturnal fasting and diminished postprandial upregulation of carbohydrate oxidation have been reported^1,16^. Despite the fact that key determinants of postprandial regulation have been addressed in a number of studies, the regulation of fasting substrate oxidation remains largely elusive. Since most individuals spend a substantial time of their day in a fasted state, the oxidized substrates following overnight fasting are probably crucial to maintain long-term health. Glucose concentrations decrease during fasting while FFA increases. We therefore hypothesize that the predominant cellular energy source after overnight fast are lipids. To test this hypothesis, and to detect disease-related alterations, we analyzed early morning fasting RQ and its´ relation to glucose and FFA in a cohort of humans, ranging from lean and healthy to obese patients with diabetes.

## Results

**Figure 1:**
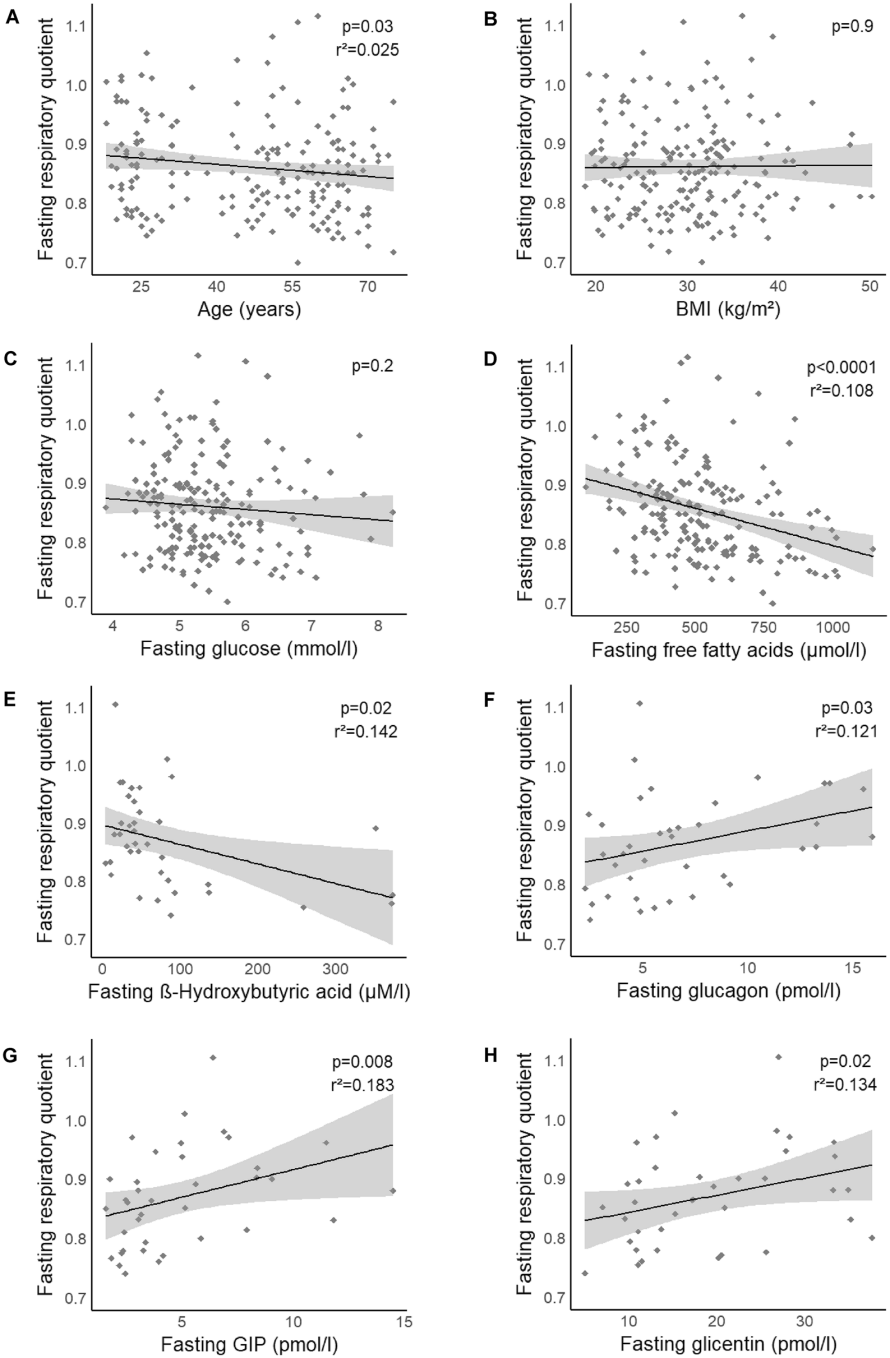
**Association of respiratory quotient (RQ) and its´ potential determinants**. RQ was negatively associated with age (**A**), with lower RQ values with increasing age. No correlation between RQ and BMI (**B**) or plasma glucose (**C**) was detected. Free fatty acids (**D**) as well as the ketone body ß-hydroxybutyric acid (**E**) were significantly correlated to RQ. Glucagon (**F**), GIP (**G**) and glicentin (**H**) were positively associated to RQ. Data are presented as scatterplots with linear regression lines and 95% CI. *p* values were taken from linear regression analyses.

**Figure 2:**
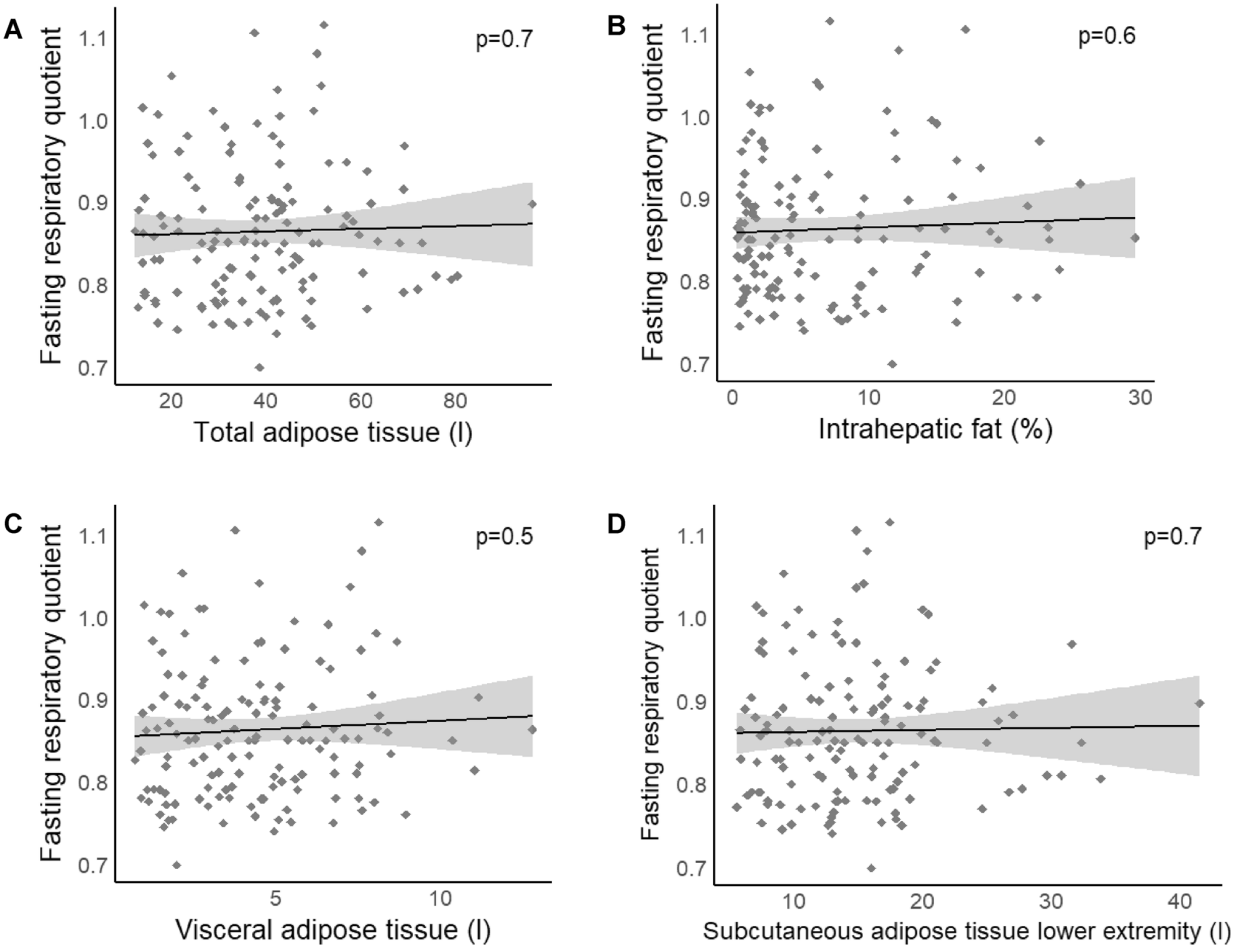
**Association of respiratory quotient (RQ) and body fat distribution determined by whole-body MRI and liver MRS**. Total adipose tissue (**A**), intrahepatic fat content (**B**), neither visceral nor subcutaneous adipose tissue (**C**, **D**) was correlated to RQ. Data are presented as scatterplots with linear regression lines and 95% CI. *p* values are derived from linear regression analyses.

In our participants, the median RQ was 0.85 (IQR: 0.79–0.9). No significant difference was found in RQ between men and women (*p* = 0.06, *p*_adj, age_ = 0.2). RQ was correlated to age, with lower values in older subjects (*p* = 0.03, *p*_adj, sex, BMI_ = 0.02; Fig. 1A). Neither BMI nor the total amount of adipose tissue was correlated to RQ (all *p* ≥ 0.06; Figs. 1B and 2A, respectively). RQ was not correlated with any of the analyzed body fat compartments (*p* ≥ 0.1; Fig. 2C,D, respectively) or with the amount of intrahepatic lipid accumulation (*p* = 0.6, *p*_adj, sex, age_ = 0.3; Fig. 2B).

We first tested whether substrate oxidation is linked to fasting substrate availability. Fasting glucose was not associated with RQ (*p* = 0.2, *p*_adj, sex, age_ = 0.7; Fig. 1C). By contrast, a significant negative correlation was found between FFA and RQ (*p* < 0.0001; Fig. 1D). This correlation remained significant after adjustment for sex and age (*p* < 0.0001). We also detected a significant negative association between ß-hydroxybutyric acid and RQ (*p* = 0.02, *p*_adj, sex, age_ = 0.02; Fig. 1E).

Since the impact of insulin is crucial for substrate availability, we further analyzed the relationship between insulin and substrate oxidation. Neither circulating insulin, nor whole-body insulin sensitivity, nor adipose tissue insulin sensitivity were related to RQ (all *p* ≥ 0.07; Table 1).

**Table 1:** Associations with respiratory quotient (RQ).

N = 192	Median (IQR)/n	*p* value (unadj./adj.*)	Stand. β (standard error)
**Sex**		0.06/0.2^#^	− 0.092 (0.0069)
Male	100		
Female	92		
Age (years)	51 (27–62)	**0.03/0.02**^##^	− 0.188 (0.017)
Body mass index (kg/m^2^)	30.1 (24.7–33.7)	0.9/0.06	0.162 (0.036)
**Blood pressure**^**a**^			
Systolic (mmHg)	137 (127–145)	0.6/0.97	− 0.003 (0.065)
Diastolic (mmHg)	89 (80–96)	0.7/0.6	0.044 (0.001)
Heart rate (bpm)^a^	69 (63–78)	0.6/0.7	− 0.029 (0.041)
**Metabolic syndrome**^**b**^		0.8/0.4	− 0.071 (0.007)
Yes	87		
No	100		
**Glycemic category**		0.6/0.8	
Normal glucose tolerance	107		
Prediabetes	75		
Diabetes mellitus (newly diagnosed, treatment naïve)	10		
**Indirect calorimetry**			
Fasting respiratory quotient	0.85 (0.79–0.90)		
Resting energy expenditure (kcal)	2008 (1746–2275)	0.3/0.9	0.009 (0.000)
**Body composition**			
Total adipose tissue, MR-derived (l)^c^	37.9 (26.5–47.3)	0.7/0.1	0.184 (0.022)
Subcutaneous adipose tissue lower extremity, MR-derived (l)^c^	13.7 (9.7–17.6)	0.7/0.1	0.161 (0.025)
Visceral adipose tissue, MR-derived (l)^d^	4.2 (2.3–6.0)	0.5/0.1	0.180 (0.017)
Intrahepatic fat, MRS-derived (%)^e^	4.1 (1.4–9.7)	0.6/0.3	0.110 (0.008)
**Glycemia**			
HbA1c (mmol/mol)^f^/HbA1c (%)^f^	37 (34–40)/5.5 (5.3–5.8)	0.9/0.3	0.090 (0.064)
Fasting glucose (mmol/l)^g^	5.3 (4.9–5.8)	0.2/0.7	− 0.033 (0.056)
Fasting insulin (pmol/l)^g^	70 (45–109)	0.5/0.1	0.122 (0.011)
Fasting C-peptide (pmol/l)^h^	516 (363–711)	0.9/0.2	0.115 (0.016)
Disposition index^i^	1243 (684–2061)	0.1/0.6	0.040 (0.009)
Insulin sensitvity index (OGTT-derived)^j^	9.6 (5.6–15.2)	0.8/0.1	− 0.119 (0.011)
Adipo-IR (mmol/l * pmol/l)^h^	36.0 (19.2–57.4)	0.07/0.3	− 0.082 (0.009)
**Plasma lipids**			
Free fatty acids (µmol/l)^h^	498 (375–633)	**< 0.0001**/**< 0.0001**	− 0.305 (0.016)
Triglycerides (mg/dl)^i^	97 (74–137)	0.8/0.4	0.073 (0.015)
Cholesterol (mg/dl)^i^	190 (158–227)	0.6/0.4	0.081 (0.000)
LDL-Cholesterol (mg/dl)^i^	121 (95–150)	0.4/0.7	0.032 (0.025)
HDL-Cholesterol (mg/dl)^i^	52 (43–62)	0.9/0.4	0.069 (0.029)
Lipoprotein (a) (mg/dl)^i^	12 (7–39)	0.3/0.3	− 0.082 (0.006)
**Proglucagon cleavage products**^k^			
Insulin/Glucagon ratio	15.5 (12.7–20.4)	0.9/0.97	0.008 (0.030)
Glucagon (pmol/l)	5.67 (4.27–8.91)	**0.03**/**0.03**	0.421 (0.032)
Glicentin (pmol/l)	17.28 (11.11–26.90)	**0.02**/**0.03**	0.365 (0.031)
Glucagon-like peptide 1 (pmol/l)	3.39 (2.22–4.25)	0.08/0.1	0.284 (0.021)
Glucose-dependent insulinotropic polypeptide (pmol/l)	3.82 (2.52–6.64)	**0.008**/**0.01**	0.474 (0.028)
**Others**			
ß-Hydroxybutyric acid (µM/l)^k^	47.2 (30.2–85.4)	**0.02**/**0.02**	− 0.382 (0.015)
Thyroid-stimulating hormone (mU/l)^i^	1.82 (1.19–2.75)	0.3/0.7	0.026 (0.009)
C-reactive protein (mg/dl)^i^	0.12 (0.03–0.35)	0.7/0.1	0.121 (0.005)
Morning cortisol, serum (nmol/l)^i^	387 (297–508)	0.5/0.2	− 0.104 (0.019)

*MR* magnetic resonance, *MRS* magnetic resonance spectroscopy, *HbA1c* hemoglobin A1c, *OGTT* oral glucose tolerance test, *Adipo-IR* adipose tissue insulin resistance index, *LDL-Cholesterol* low-density lipoprotein cholesterol, *HDL-Cholesterol*: high-density lipoprotein cholesterol.

*Adjusted for sex and age; # adjusted for age; ## adjusted for sex and BMI. Standardized ß are from multivariate linear regression models. ^a^: n = 190; ^b^: n = 187; ^c^: n = 137; ^d^: n = 139; ^e^: n = 140; ^f^: n = 178; ^g^: n = 191; ^h^: n = 181; ^i^: n = 179; ^j^: n = 184; ^k^: n = 38. p < 0.05 are printed in bold.

We next analyzed the relationship of proglucagon cleavage products and GIP to RQ. Albeit there was no link for GLP-1 (*p* = 0.08, *p*_adj, sex, age_ = 0.1; Table 1), glucagon (*p* = 0.03; Fig. 1F), glicentin (*p* = 0.02; Fig. 1H) and GIP (*p* = 0.008; Fig. 1G) were positively associated with RQ. These associations remained significant after adjustment for sex and age (*p* ≤ 0.03). Following adjustment for FFA, glucagon was no longer associated with RQ (*p*_adj, sex, age, FFA_ = 0.09), whereas the association of RQ and GIP as well as with glicentin remained significant (*p* ≤ 0.04). Moreover, adjustment for fasting glucose did not alter the association of glucagon with RQ.

Neither thyroid-stimulating hormone nor serum cortisol was correlated to RQ (all *p* ≥ 0.2; Table 1).

We further addressed the relationship between metabolic disorders and fasting substrate oxidation. RQ was comparable between participants with normal glucose tolerance, prediabetes or newly-diagnosed type 2 diabetes (*p* = 0.6, *p*_adj, sex, age_ = 0.8; Table 1). It was also comparable between participants with and without the metabolic syndrome (*p* = 0.8, *p*_adj, sex, age_ = 0.4; Table 1).

We next tested for potential interactions between weight groups (normal weight/overweight vs obese) and anthropometric/clinical parameters on RQ. These analyses revealed no statistically significant interactions for glucose and FFA, indicating that their relation to RQ is comparable between the two weight groups (Suppl. Table 1). Similar interaction analyses with glycemic categories and presence/absence of the metabolic syndrome also revealed no interactions between glucose or FFA on RQ (interaction with glycemic categories: *p*_(interaction glycemic cat × glucose)_ = 0.2 and *p*_(interaction glycemic cat × FFA)_ = 0.8; interaction with metabolic syndrome: *p*_(interaction met syndrome × glucose)_ = 0.5 and *p*_(interaction met syndrome × FFA)_ = 0.1).

No association was found between systolic or diastolic blood pressure and RQ (all *p* ≥ 0.6). Since 53 of the 192 participants received antihypertensive medication, we repeated the analysis after excluding the former. Thereafter, blood pressure still did not correlate with RQ (all *p* ≥ 0.4). However, the correlation between FFA and RQ remained significant (*p* = 0.003, *p*_adj, sex, age_ = 0.005).

We next analyzed resting energy expenditure. There was a negative correlation of age with resting energy expenditure (Suppl. Fig. 1A). Higher BMI was associated with higher resting energy expenditure (Suppl. Fig. 1B). While resting energy expenditure was positively associated with fasting glucagon (Suppl. Fig. 1F), this significance disappeared after adjustment for sex (*p* = 0.7).

## Discussion

We investigated potential major metabolic determinants of substrate oxidation in the fasting state. An analysis of a wide range of participants, from lean and healthy to obese with diabetes, enabled us to identify FFA as a potential major determinant of substrate oxidation. Furthermore, glicentin, GIP and ketone bodies were additional independent determinants. By contrast, glycemia was not related to nutrient utilization. Surprisingly, no link could be established between the preferred oxidized substrate and metabolic diseases, including obesity, diabetes and the metabolic syndrome.

In contrast to our hypothesis, the predominant cellular energy source before breakfast in the morning was not solely FFA, but a mixture of substrates with large intra-individual differences (and a RQ range of 0.7–1.1). This tallies well with previous findings in a number of populations^17^.

Aging has been reported to result in a shift from fat to glucose oxidation in some^18,19^ but not all previous trials^20^. Our analysis detected higher rates of fat oxidation in older persons. Age-related insulin resistance has been proposed to be one contributing factor^21^. However, since we did not detect any association between insulin sensitivity and RQ, our results do not support this hypothesis. Since substrate oxidation takes place in the mitochondria, age-related alterations in mitochondrial function^22^, together with rising FFA concentrations, could govern shifts in substrate oxidation towards lipids during aging. Results on differences in substrate utilization between sexes are inconsistent^6,12,18,20,23^. We did not detect any significant differences in fasting RQ.

Glucose is a major energy source, and a sufficient supply is ensured even in the fasting state. While more ATP is generated from lipids, cellular energy formation from glucose is more efficient in terms of oxygen consumption^24^, and some cell types largely depend on glucose. In the muscle, there is a competition between glucose and FFA as the major energy source^25^. However, there is no link between circulating glucose and substrate utilization. While carbohydrate availability has been proposed as a determinant^26^, our current results argue against the hypothesis that substrate oxidation is purely the result of substrate availability.

In contrast to glucose, circulating FFA are robustly linked to cellular substrate oxidation, independent of the tested confounders. When abundantly available or experimentally elevated^27^, FFA tend to be oxidized. This tallies with earlier reports^28,29^ and is in accordance with Randle’s hypothesis that fatty acids suppress glucose oxidation in skeletal muscle^25^. Indeed, fatty acid oxidation is directly linked to circulating FFA concentrations^30^. Of note, the regulation of substrate oxidation by FFA was independent of insulin, insulin-sensitivity and of the amount of lipids stored in adipose tissue. This might be due to direct intracellular regulation, since fatty acids and their metabolic intermediates are not only energy sources but also possess signaling properties^31,32^.

Unlike prior reports^14,29,33^, we found no connection between fasting insulin or insulin sensitivity and substrate oxidation. Inclusion of participants with different glycemic categories cannot explain these differences, as glycemic status did not influence our detected relations. Insulin is a potent suppressor of lipolysis and thereby inhibits FFA supply. Concurrently, it stimulates cellular glucose uptake and could thereby promote glucose oxidation. However, these functions occur in the postprandial state, during which insulin concentrations rise rapidly. Theoretically, in the fasting state, insulin should not determine substrate oxidation. This could explain why we did not detect any links to RQ, while other studies which included postprandial periods found such contributions of insulin.

Some organs, including the brain, are unable to utilize fatty acids as their sole energy source. These organs rely on ketone bodies, formed from fatty acids in the liver, as an alternative energy source^34^. In the current study, we assessed ß-hydroxybutyric acid which is, quantitatively speaking, the major ketone body. The link of ketone bodies with lower RQ is plausible since their formation depends on fatty acid oxidation in the liver. Furthermore, the utilization of ketone bodies also results in a low RQ. Physiologic ketone body production has recently been linked to beneficial effects on heart, brain and muscle^35^. High spontaneous ketones were even found to be predictive of long-term reduced risk for diabetes^36^.

Further novel observations are the correlations between substrate oxidation and GIP as well as with glicentin. Upon food intake, the incretins GIP and glicentin are secreted from duodenal cells and subsequently modulate energy metabolism^37,38^. Their regulation in the fasting state is largely unknown. We observed lower rates of fat oxidation in persons with higher fasting levels of circulating GIP and glicentin. Our data are in line with animal models in which GIP signaling decreased fat oxidation via skeletal muscle and adipose tissue^39^ and was crucial in cellular lipid handling^40^. How glicentin contributes to substrate oxidation is still unknown and further experimental studies are required to determine the significance of our findings.

The detected association of glucagon and RQ was presumably mediated by FFA, which is probably due to glucagon’s stimulatory effect on FFA turnover^41^. We assume that glucagon reduces the availability of FFA since the latter serve as precursors for gluconeogenesis. This could explain the observed increased rates of glucose oxidation in persons with high circulating glucagon levels.

Several trials reported a relationship between fat mass and lipid oxidation^33,42,43^. As visceral fat associates with insulin resistance and lower fat oxidation^44,45^, whereby subcutaneous fat is linked to higher fat oxidation^46,47^, we quantified both. However, fuel selection was associated with neither BMI, nor fat mass, nor body fat distribution. This is in agreement with Ferro et al., who likewise detected no link between fasting RQ and body fat content^12^. Our data therefore do not support the hypothesis of reduced lipid oxidation as a cause or consequence of obesity. This “low fat oxidation hypothesis” was discussed in a recent meta-analysis which came to the conclusion that there is no convincing experimental evidence to support this hypothesis^17^.

While one study reported lower fasting RQ in diabetes^10^, neither we nor two earlier trials^12,20^ detected such a difference to persons with normal glucose tolerance. Thus, fasting substrate oxidation does not appear to make any major contribution to body weight, body fat distribution, or metabolic diseases.

Since our current work is a cross-sectional analysis, the data do not permit us to test the prospective value of RQ. Furthermore, some associations might only be present in specific subgroups that were not available in sufficiently large numbers to warrant analysis in our study. The fasting concentrations of some of the analyzed hormones are rather low and their physiological relevance in the fasting state has not been extensively studied so far (e.g. incretins).

Taken together, we detected FFA as a potential major determinant of fasting substrate oxidation and possible contributions of glicentin and GIP as well as of ketone bodies. Since the preferred oxidized substrate was not linked to metabolic diseases, fuel selection in the fasting state probably does not make any major contribution to obesity, diabetes, or the metabolic syndrome. Upcoming pharmacological approaches target GIP signaling^48,49^. Our results indicate that they have the potential to modulate preferred substrate oxidation.

## Materials and methods

Data acquisition was performed at the University Hospital of Tübingen between October 2016 and August 2020, as part of ongoing clinical studies (NCT02991365, NCT03590561, NCT03615209, NCT03227484, NCT03525002, and NCT04052399). The study protocols were approved by the local ethics committee (Ethics Committee of the Medical Faculty of the Eberhard Karls University and the University Hospital Tübingen) and all experiments were conducted in accordance with the relevant guidelines and regulations. Further, all participants provided informed written consent.

### Study design and participants

One hundred and ninety-six participants from six ongoing trials were included in this cross-sectional analysis. We selected all participants, for whom data from indirect calorimetry in the fasting state were available, which was performed at the beginning of each study. Four participants were excluded from data analysis due to implausible indirect calorimetry or laboratory measurements. Participants had a median age of 51 years (IQR: 28–62), the majority were overweight or obese (median (IQR): BMI: 30.1 kg/m^2^ (25.0–33.6)), and had a median HbA1c of 5.5% (5.3–5.8). 53 participants took antihypertensive medication. Patient characteristics are reported in Table 1 and Supplementary Table 2.

A medical history was recorded and a physical examination was performed. Following an overnight fast and an indirect calorimetry, all participants were subjected to a 5-point, 75-g oral glucose tolerance test (OGTT) to assess insulin sensitivity, insulin secretion and glycemic categories. Normal glucose tolerance was found in 107 participants, 75 had prediabetes, and in 10 patients, type 2 diabetes was diagnosed by way of this OGTT. None of the participants took any medication interfering with glucose metabolism.

### Laboratory measurements and calculations

Venous blood samples were taken before, as well as 30, 60, 90 and 120 min after glucose administration. Serum insulin and C-peptide were determined by an immunoassay on an ADVIA Centaur system (Siemens Healthineers, Eschborn, Germany). Glucose and triglycerides, together with total, HDL, and LDL lipoprotein cholesterol levels were assessed using the ADVIA XPT clinical chemical system (Siemens Healthineers, Eschborn, Germany). Plasma concentrations of total FFA were measured with an enzymatic method (WAKO Chemicals, Neuss, Germany) on the latter instrument. Glycated hemoglobin (HbA1c) measurements were carried out with the Tosoh A1c analyzer HLC-723G8 (Tosoh Bioscience GmbH, Griesheim, Germany).

Whole-body insulin sensitivity was determined using Matsuda Index^50^. One unusually high value for Matsuda Index (> 5 SD above median) was excluded from data analysis. Insulin resistance of adipose tissue at fasting was estimated by Adipo-IR Index (adipose tissue insulin resistance index)^51^. Insulin secretion was assessed by Disposition Index^52^.

Metabolic syndrome was classified in accordance with the criteria of the International Diabetes Federation (IDF)^53^, while glucose tolerance was in accordance with the criteria of the American Diabetes Association (ADA)^54^.

In a subset of 38 participants, fasting glucagon, GLP-1, GIP, and glicentin were measured by commercial immunoassays (Mercodia, Uppsala, Sweden). Fasting β-hydroxybutyric acid was measured by an enzymatic 3-hydroxybutyrate dehydrogenase-based Ketone Body Assay (Sigma-Aldrich, St. Louis, MO, USA).

### Indirect calorimetry

Indirect calorimetry was performed in the morning after an overnight fast of at least 10 h abstinence from food, nicotine and caffeine.

The exhaled air was analyzed with Vyntus CPX (Carefusion, Hoechberg, Germany). Total average energy expenditure in kilocalories (kcal) was calculated using the modified Weir´s equation^55^.

During substrate oxidation, oxygen (VO_2_) is consumed and carbon dioxide (VCO_2_) is produced. The ratio of VCO_2_ and VO_2_ in the exhaled air represents the respiratory quotient. It is dependent on the relative amounts of oxidized substrates and reflects substrate utilization. Physiologically, the RQ ranges between 0.67 and 1.2^56,57^. Under stable protein utilization, an RQ equal to 1 indicates preferential glucose metabolism, while an RQ of 0.7 reflects predominantly lipid oxidation.

### Body fat distribution and intrahepatic fat

Quantification of whole-body adipose tissue and intrahepatic fat was performed on the same day prior to indirect calorimetry by magnetic resonance imaging (MRI) and proton magnetic resonance spectroscopy (^1^H-MRS). A 3T whole-body imager (Magnetom Vida, Siemens Healthineers, Erlangen, Germany), as described previously^58^, was used. Segmentation of adipose tissue was performed by an automatic procedure based on fuzzy clustering^59^. Intrahepatic fat was quantified by localized ^1^H-MRS from the posterior part of segment 7 by applying a single-voxel STEAM technique with a short echo time (TE = 10 ms). Integrals of methylene/methyl resonances (lipids) and water were assessed and intrahepatic fat was determined in percent by calculating the ratio of lipids and water + lipids.

### Statistics

Statistical analysis was carried out using JMP 14.0 (SAS Institute, Cary, NC, USA). Prior to analysis, skewed variables were log transformed. Correlations were tested by multivariate linear regression analysis with adjustments for sex and age. Data are presented as median with IQR. *p* values < 0.05 were considered as statistically significant.

## Supplementary Information

Supplementary Information.

## Data Availability

The data are not publicly available due to them containing information that could compromise research participant privacy/consent.
