# Supplementary material for: Free fatty acids, glicentin and glucose-dependent insulinotropic polypeptide as potential major determinants of fasting substrate oxidation
Source: Sci Rep. 2021 Aug 17;11:16642. doi: 10.1038/s41598-021-95750-9 (PMC8371005; doi:10.1038/s41598-021-95750-9)
Supplement: Supplementary file 1 — Supplementary Information. [file 41598_2021_95750_MOESM1_ESM.pdf]

Supplementary material

Free fatty acids, glicentin and glucose-dependent insulinotropic polypeptide as potential major determinants of fasting substrate oxidation

Julia Hummel[1,2], Louise Fritsche[1,2], Andreas Vosseler[1-3], Corinna Dannecker[1,2], Miriam Hoene[4], Konstantinos Kantartzis[1-3], Hans-Ulrich Häring[1-3], Norbert Stefan[1-3], Jürgen Machann[1,2,5], Andreas L. Birkenfeld[1-3], Cora Weigert[1,2,4,] Robert Wagner[1-3], Andreas Peter[1,2,4], Andreas Fritsche[1-3], Martin Heni[1-4]

1. Institute for Diabetes Research and Metabolic Diseases of the Helmholtz Center
1. Munich at the University of Tübingen, Otfried-Müller-Str. 10, 72076 Tübingen, Germany
2. German Center for Diabetes Research (DZD), Ingolstädter Landstraße 1, 85764 Neuherberg, Germany
3. Department of Internal Medicine, Division of Diabetology, Endocrinology and Nephrology, Eberhard Karls University Tübingen, Otfried-Müller-Str. 10, 72076 Tübingen, Germany
4. Institute for Clinical Chemistry and Pathobiochemistry, Department for Diagnostic Laboratory Medicine, Eberhard Karls University Tübingen, Hoppe-Seyler-Str. 3, 72076 Tübingen, Germany
5. Department of Radiology, Section on Experimental Radiology, Eberhard Karls University Tübingen, Hoppe-Seyler-Str. 3, 72076 Tübingen, Germany

Supplementary table 1: Associations with respiratory quotient (RQ) stratified by weight group.

N = 192	**Lean/Overweight** (BMI < 30 kg/m²) N=95			**Obese** (BMI ≥ 30 kg/m²) N=97			
	**Median (IQR) / n**	**p value** (unadj./adj.*)	**Stand. β** (standard error)	**Median (IQR) / n**	**p value** (unadj./adj.*)	**Stand. β** (standard error)	$p_{interaction}$
Sex		0.2 / 0.5[#]	-0.078 (0.012)		0.1 / 0.06[#]	-0.191 (0.010)	0.9
Male	65			62			
Female	30			35			
Age (years)	31 (23 - 61)	0.1 / 0.4[##]	-0.113 (0.022)	54 (44 - 62)	**0.03** / **0.02**[##]	-0.245 (0.031)	0.3
Body mass index (kg/m²)	24.6 (21.9 - 27.8)	0.7 / 0.3	0.141 (0.099)	33.6 (32.0 - 37.9)	0.6 / 0.7	0.042 (0.077)	0.5
Blood pressure							
Systolic (mmHg)	132 (125 – 142)[a]	0.2 / 0.1	0.173 (0.101)	140 (133 – 148)	0.07 / 0.1	-0.178 (0.087)	**0.03**
Diastolic (mmHg)	84 (77 – 90)[a]	1.0 / 0.6	0.064 (0.001)	91 (85 – 100)	0.5 / 0.9	-0.016 (0.001)	0.6
Heart rate (bpm)	66 (61 – 75)[a]	0.2 / 0.2	-0.152 (0.059)	72 (65 – 81)	0.7 / 0.7	0.039 (0.060)	0.2
Metabolic syndrome		1.0 / 0.4	-0.096 (0.013)		0.7 / 0.7	0.038 (0.010)	0.8
Yes	19[a]			68[b]			
No	74[a]			26[b]			
Glycemic category		0.7 / 0.2			0.1 / 0.2		0.2
Normal glucose tolerance	66			41			
Prediabetes	28			47			
Diabetes mellitus (newly diagnosed, treatment naïve)	1			9			
**Indirect calorimetry**							
Fasting respiratory quotient	0.85 (0.79 – 0.90)			0.85 (0.80 – 0.90)			
Resting energy expenditure (kcal)	1925 (1649 – 2211)	0.3 / 1.0	-0.004 (0.073)	2108 (1839 – 2454)[c]	0.9 / **0.03**	-0.310 (0.071)	0.4

**Body composition**							
Total adipose tissue, MR-derived (l)	23.6 (16.4 – 30.9)[d]	0.8 / 0.9	-0.040 (0.058)	44.5 (41.1 – 55.7)[e]	0.8 / 0.8	0.027 (0.046)	0.7
Subcutaneous adipose tissue lower extremity, MR-derived (l)	9.2 (7.5 – 12.8)[d]	0.9 / 0.9	0.025 (0.057)	16.9 (14.1 – 20.1)[e]	0.9 / 1.0	-0.000 (0.040)	0.9
Visceral adipose tissue, MR-derived (l)	2.1 (1.5 – 3.5)f	0.95 / 0.8	0.049 (0.031)	5.1 (4.2 – 7.4)e	0.7 / 1.0	-0.003 (0.034)	0.8
Intrahepatic fat, MRS-derived (%)	1.4 (0.8 – 3.1)[g]	0.6 / 0.6	0.075 (0.013)	7.1 (3.4 – 14.2)[h]	0.9 / 0.8	-0.023 (0.011)	0.7
**Glycemia**							
HbA1c (mmol/mol) / HbA1c (%)	36 (33 – 38)[i] 5.4 (5.2 – 5.7)[i]	0.9 / 0.2	0.160 (0.190)	39 (36 – 42)[j] 5.7 (5.4 – 6)[j]	0.7 / 1.0	-0.000 (0.131)	0.8
Fasting glucose (mmol/l)	5.1 (4.7 – 5.5)[b]	0.8 / 0.3	0.137 (0.093)	5.6 (5.1 – 6.2)[k]	**0.04** / **0.04**	-0.124 (0.072)	0.1
Fasting insulin (pmol/l)	53 (36 – 70)[b]	0.8 / 0.5	0.082 (0.019)	99 (70 – 144)[k]	0.4 / 0.7	0.043 (0.017)	0.7
Fasting C-peptide (pmol/l)	394 (270 – 573)[a]	0.5 / 0.1	0.185 (0.024)	666 (501 – 853)[j]	0.9 / 0.8	-0.025 (0.026)	0.6
Disposition index	1751 (953 – 2735)[j]	0.6 / 0.8	-0.026 (0.014)	1001 (579 – 1470)[l]	0.09 / 0.2	0.146 (0.012)	0.4
Insulin sensitvity index (OGTT-derived)	14.4 (10.2 – 19.2)[m]	0.8 / 0.3	-0.130 (0.021)	6.3 (4.4 – 9.2)[n]	0.7 / 0.9	-0.012 (0.017)	1.0
Adipo-IR (mmol/l*pmol/l)	22.4 (14.5 – 39.5)[a]	0.09 / 0.2	-0.135 (0.014)	49.9 (32.9 – 79.7)[j]	0.3 / 0.3	-0.108 (0.014)	0.7
**Plasma lipids**							
Free fatty acids (µmol/l)	464 (319 – 654)[a]	**0.005** / **0.01**	-0.269 (0.020)	511 (407 – 632)[j]	**<0.001** / **<0.001**	-0.370 (0.028)	0.1
Triglycerides (mg/dl)	73 (54 – 88)[o]	0.9 / 0.6	0.051 (0.025)	108 (82 – 141)[e]	0.6 / 0.7	0.050 (0.022)	0.8
Cholesterol (mg/dl)	172 (147 – 206)[l]	0.6 / 0.5	0.104 (0.000)	210 (177 – 234)[j]	0.9 / 0.7	0.039 (0.000)	0.8
LDL-Cholesterol (mg/dl)	104 (83 – 131)[l]	0.7 / 0.6	0.062 (0.034)	141 (114 – 165)[j]	0.4 / 0.7	-0.051 (0.043)	0.6
HDL-Cholesterol (mg/dl)	55 (44 – 67)[l]	0.7 / 0.7	0.039 (0.042)	49 (43 – 59)[j]	0.5 / 0.1	0.168 (0.045)	0.4

Lipoprotein (a) (mg/dl)	11 (6 – 42)[l]	0.4 / 0.4	-0.096 (0.009)	14 (7 – 32)[j]	0.5 / 0.5	-0.067 (0.010)	1.0
**Others**							
Thyroid-stimulating hormone (mU/l)	2.0 (1.4 – 3.1)[l]	0.7 / 0.2	-0.14 (0.016)	1.7 (1.1 – 2.4)[j]	0.1 / 0.2	0.137 (0.011)	0.2
C-reactive protein (mg/dl)	0.04 (0.01 – 0.15)[l]	0.6 / 0.2	0.141 (0.007)	0.26 (0.11 – 0.56)[j]	0.8 / 0.9	0.020 (0.008)	0.9
Morning cortisol, serum (nmol/l)	413 (336 – 524)[l]	0.7 / 1.0	0.005 (0.027)	368 (277.8 – 488.8)[j]	0.2 / 0.06	-0.210 (0.026)	0.2

*Adjusted for sex and age; # adjusted for age; ## adjusted for sex. Standardized ß are from multivariate linear regression models. a: n=93; b: n =94; c: n=96; d: n=57; e: n=80, f: n=59; g: n=61; h: n=79; i: n=90; j: n=88; k: n=79; l: n=91; m: n=89; n: n=95; o: n= 71. MR: magnetic resonance; MRS: magnetic resonance spectroscopy; HbA1c: hemoglobin A1c; OGTT: oral glucose tolerance test; Adipo-IR: adipose tissue insulin resistance index; LDL-Cholesterol: low-density lipoprotein cholesterol; HDL-Cholesterol: high-density lipoprotein cholesterol.

Supplementary figure 1

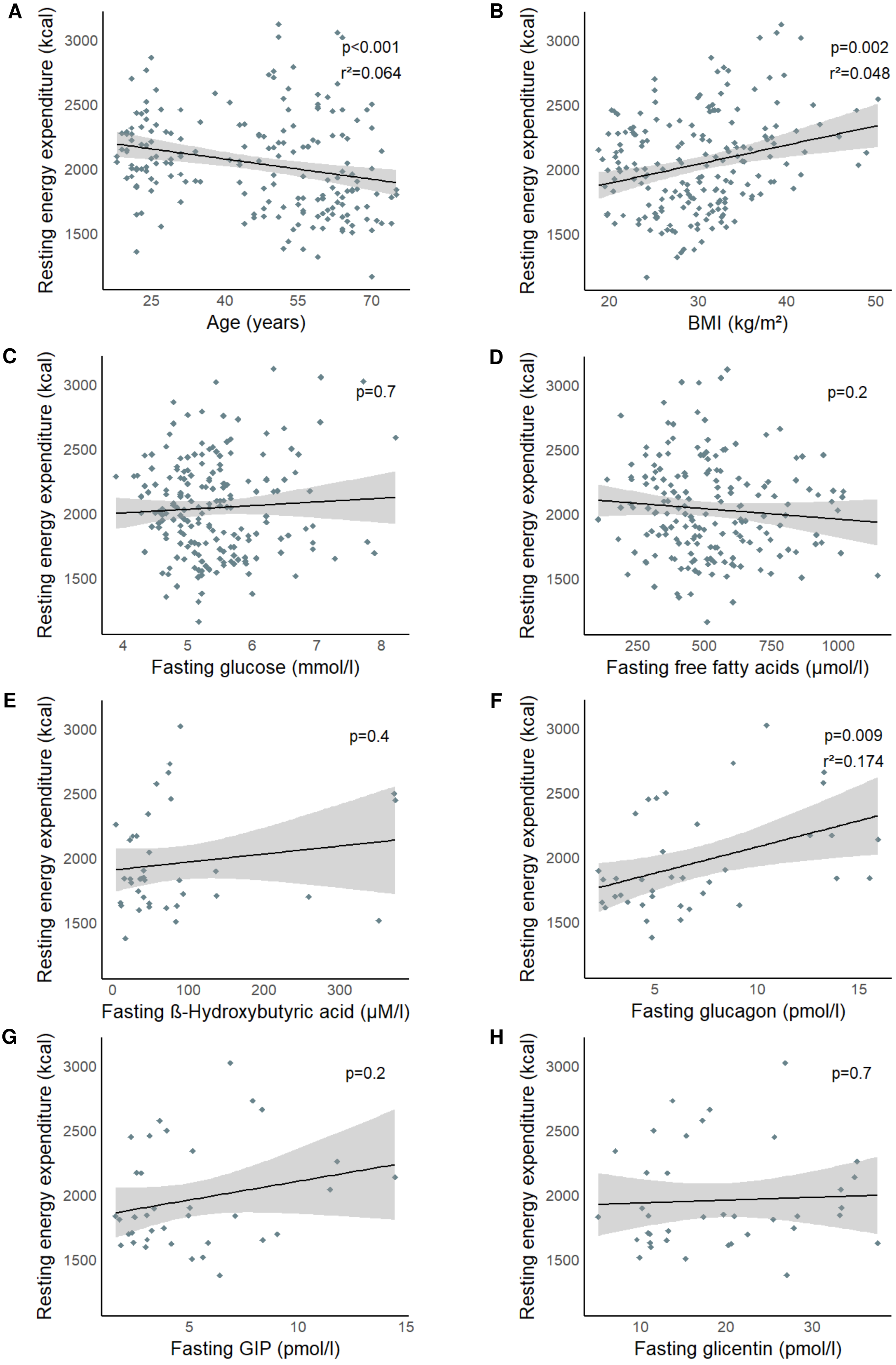

Supplementary figure 1 - Association resting energy expenditure and its´ potential determinants

Resting energy expenditure was negatively associated with age **(A)**, with lower resting energy expenditure with increasing age. There was a positive association of resting energy expenditure and BMI **(B).** Resting energy expenditure was neither significantly correlated with plasma glucose **(C)** nor with free fatty acids **(D)** nor with the ketone body ß-hydroxybutyric acid **(E)**. While glucagon was positively associated with resting energy expenditure **(F)**, this association was not independent of sex (p after adjustment for sex 0.7). Neither GIP **(G)** nor glicentin **(H)** were associated with resting energy expenditure. Data are presented as scatterplots with linear regression lines and 95% CI. P-values were taken from linear regression analyses.

Supplementary table 2: Patient characteristics

	Overall cohort (n=192)	Sub-group with incretin measurements (n=38)
	Median (IQR) / n	
Sex		
Male	100	16
Female	92	22
Age (years)	51 (27 – 62)	62 (56 – 66)
Body mass index (kg/m^2)	30.1 (24.7 – 33.7)	31.5 (28.7 – 33.3)
Total adipose tissue, MR-derived (l)	37.9 (26.5 – 47.3)[a]	39.8 (32.2 – 42.9)
HbA1c (mmol/mol)/<br>HbA1c (%)	37 (34 – 40)[b]<br>5.5 (5.3 – 5.8)[b]	39 (37 – 41)<br>5.7 (5.5 – 5.9)
Fasting glucose (mmol/l)	5.3 (4.9 – 5.8)[c]	5.9 (5.6 – 6.5)
Fasting insulin (pmol/l)	70 (45 – 109)[c]	91 (62 – 147)
Insulin sensitvity index (OGTT-derived)	9.6 (5.6 – 15.2)[d]	5.9 (4.0 – 8.8)
Free fatty acids (µmol/l)	498 (375 – 633)[e]	519 (430 – 651)
Triglycerides (mg/dl)	97 (74 – 137)[f]	98 (73 – 139)[g]
Fasting respiratory quotient	0.85 (0.79 – 0.90)	0.86 (0.80 – 0.92)

Data are presented as median (IQR); a: n=137; b: n =178; c: n=191; d: n=184; e: n=181; f: n=179; g: n=34. MR: magnetic resonance; HbA1c: hemoglobin A1c; OGTT: oral glucose tolerance test.
